# Endophthalmitis caused by *Mycobacterium houstonense*: case report

**DOI:** 10.1186/s12879-020-05590-7

**Published:** 2020-11-16

**Authors:** Xin Che, Qingjian Li, Luoziyi Wang, Jing Jiang, Xuzhong Shen, Yiwen Qian, Zhiliang Wang

**Affiliations:** Department of Ophthalmology, Huashan Hospital of Fudan University, No. 12, Middle Urumqi Road, Shanghai, 200040 China

**Keywords:** *Mycobacteria houstonense*, Rapidly growing mycobacteria (RGM), Glaucoma drainage implant, Conjunctival erosion, Endophthalmitis

## Abstract

**Background:**

*Mycobacterium houstonense* is rapidly growing mycobacteria (RGM) that belongs to *M. fortuitum* group. So far, there have been few associated reports of human diseases induced by *M. houstonense* worldwide.

**Case presentation:**

We present a delayed-onset postoperative endophthalmitis caused by *M. houstonense* after glaucoma drainage implant (GDI) surgery. The ocular infection lasted for 2 months without appropriate treatment that developed into endophthalmitis and the patient underwent an emergency enucleation.

**Conclusion:**

Implant erosion and a delay in diagnosis of ocular infection could lead to irreversible damage as observed in our case. Ophthalmologists should be alert for ocular RGM infection, and prompt laboratory diagnosis with initiation of effective multidrug therapy might prevent loss of vision.

## Background

Currently, the species of rapidly growing mycobacteria (RGM) capable of producing disease in humans are grouped into six major taxonomic groups according to pigmentation and genetic relatedness. The major taxa are the *Mycobacterium fortuitum* group, *M. chelonae/M. abscessus* complex, *M. smegmatis* group, *M. mucogenicum* group, *M. mageritense/M. wolinskyi*, and the pigmented RGM. *M. fortuitum* group has historically included *M. fortuitum*, *M. peregrinum*, *M. senegalense*, *M. porcinum*, *M. neworleansense*, *M. boenickei*, *M. houstonense*, *M. brisbanense*, *M. septicum*, and *M. setense* [1].

Ocular infections caused by RGM are keratitis, scleritis, uveitis and endophthalmitis. The predisposing factors of ocular RGM infections include trauma, cataract surgery, intravitreal injection, corticosteroid use, endogenous endophthalmitis, glaucoma drainage implant (GDI) and systemic immunosuppression [2]. Endophthalmitis induced by RGM is an uncommon condition encountered post ocular surgery or trauma, which is sight threatening, may be acquired through contamination of water or antiseptic solutions. Most RGM endophthalmitis cases reported in the literature are exogenous, most of which occurred following cataract surgery [3, 4], multiple corneal transplantation [5, 6], intravitreal triamcinolone injection [7], ocular trauma [8], GDI surgery [9], silico ne-filled eye [10].

Here, we present a case of delayed-onset postoperative endophthalmitis caused by *M. houstonense* after GDI surgery. The device of GDI is used to drain aqueous humor from the anterior chamber of the eye into the subconjunctival or suprachoroidal space in order to lower the intraocular pressure in glaucoma cases [11].

## Case presentation

A 45-year-old man presented to our eye clinic with complains of unbearable pain in the left eye and headache. The patient had history of GDI surgery 3 years ago in his left eye with Ahmed glaucoma valve (AGV) implantation. He experienced pain of the left eye in early February, went to see doctors in mid-February with treatment of intravenous clindamycin 1.2 g/qd for more than 10 days, the medication could temporarily relive the ocular pain. Due to COVID-19 pandemic, the patient did not consult doctors again, he took counter paracetamol by himself to relive pain. One month later he noticed displacement of AGV from his left eye, and then came to our eye clinic. The patient did not have any underlying diseases or medication history, his work was on freshwater aquaculture.

**Fig. 1:**
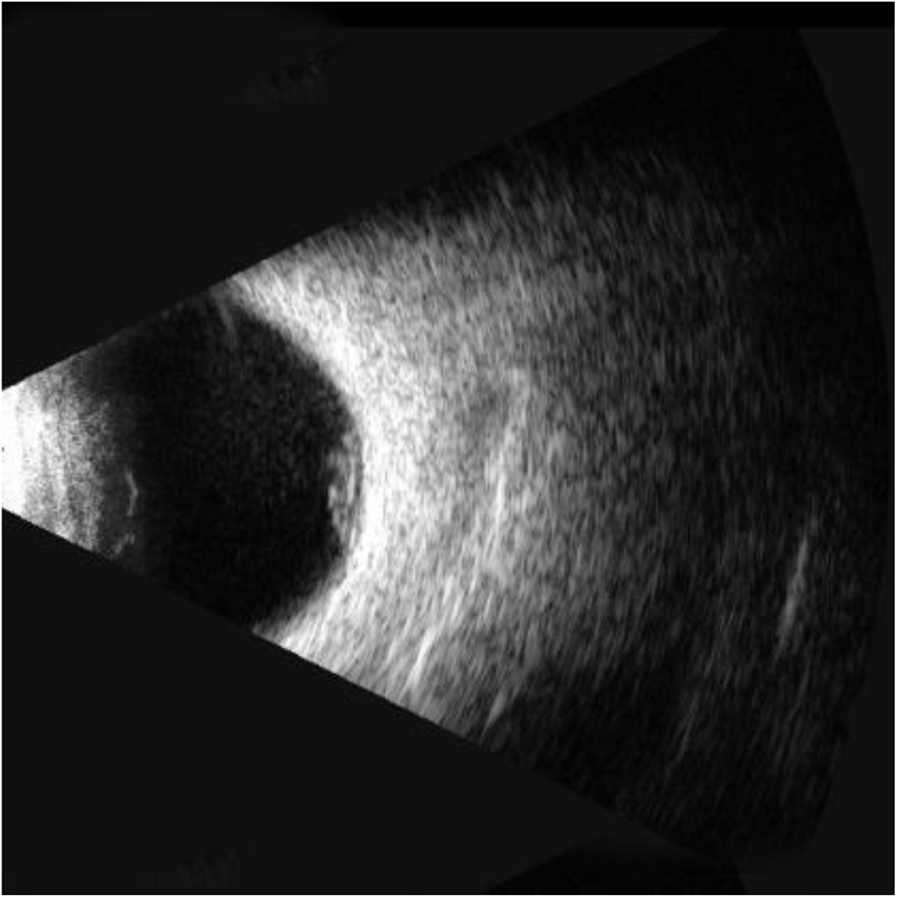
B-ultrasound of the left eye showing mild vitreous opacity, and highly reflective masses protruding into the vitreous cavity

On ophthalmic examination, the best-corrected visual acuity (BCVA) was 20/20 (OD) and no light perception (OS), with intraocular pressure (IOP, normal range 10–21 mmHg) of 17 mmHg (OD) and Tn (normal tension) + 1 (OS). The left eye showed conjunctival edema with congestion, corneal edema, hypopyon, and ocular fundus was not visible microscopically. No obvious abnormality was found in the right eye. The blood count revealed mild leukocytosis. Ocular ultrasonography showed vitreous opacity and mild atrophy of the left eye (Fig. 1). Thus the condition was presumptively diagnosed as infectious endophthalmitis. Considering the infection has resulted in severe intraocular tissue destruction and deteriorated vision, emergency enucleation of the infected eyeball was performed to prevent dissemination of the infection and sympathetic ophthalmia [12, 13]. During the surgery purulent exudates were observed, the ocular specimens and purulent exudates were subjected to microbiological investigation.

**Fig. 2:**
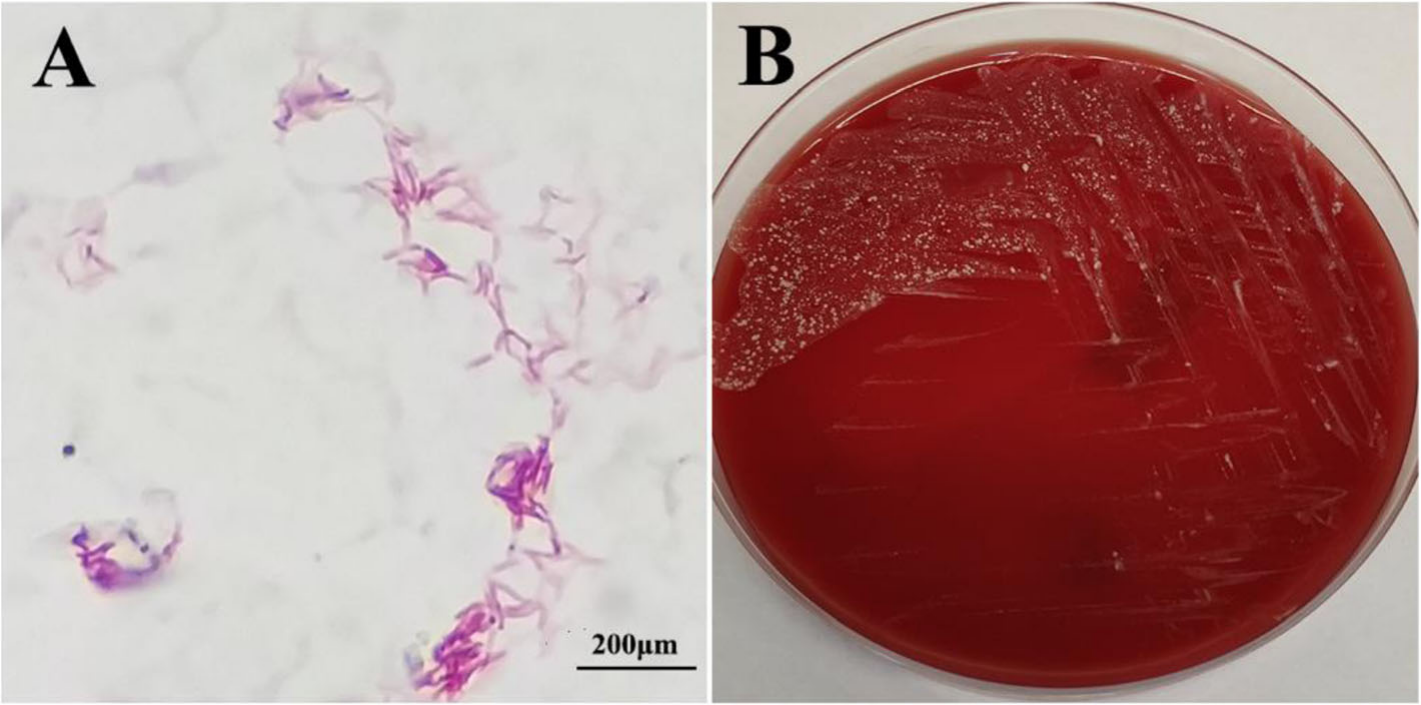
**a** Ziehl–Neelsen staining showing acid-fast bacilli isolated from the ocular purulent exudates. **b** Monoclonal colonies on blood agar plates, which are round, smooth and beige color on the second day of incubation

Within 48 h, bacterial growth was observed in blood agar when cultured in 5% CO2, at 35 °C (Fig. 2b). Ziehl-Neelsen staining of the monoclonal colonies revealed the presence of acid-fast bacilli (Fig. 2a), which was further identified as *Mycobacterium houstonense* by 16S rRNA and 16S–23S rRNA PCR, the primers are listed in Table 1. The amplified sequences of 16S rRNA and 16S–23S rRNA spacer region gene were compared with those available in the National Center for Biotechnology Information GenBank database, which revealed homology of 99.86 and 98.7% with *M. houstonense* (GenBank sequence ID: KJ913784.1) respectively.

**Table 1:** Primers targeting16S rRNA and 16S–23S rRNA of *M. houstonense* for PCR

	Forward	Reverse
16S rRNA	AGAGTTTGATCMTGGCTCAG	GGTTACCTTGTTACGACTT
16S–23S rRNA	TTGTACACACCGCCCGTCA	TCTCGATGCCAAGGCATCCACC

Antimicrobial susceptibility against *M. houstonense* was determined using minimal inhibitory concentration (MIC) by micro broth dilution method, the procedure and interpretation of the results were conducted in accordance with the CLSI M100 guidelines [14, 15]. The results showed that the strain was susceptible to levofloxacin, ciprofloxacin and amikacin, which was resistant to doxycycline, sulfamethoxazole and tobramycin. Subsequently, the patient was treated with a combination of intravenous amikacin 0.6 g/qd and oral levofloxacin 0.5 g/qd according to the drug sensitivity results for a week, and then microbiological investigations on conjunctival sac secretion and the blood were tested negative. Subsequently the patient was discharged to see the infectious specialist, and the treatment regimen was changed to oral levofloxacin 0.5 g twice a day for 40 days, the patient was followed up for 4 months without any systemic and ocular abnormality.

## Discussion

RGM is a rare cause of endophthalmitis. To date, our case was the first to demonstrate an ocular *M. houstonense* infection without any signs of systemic association. The patient had history of GDI surgery for refractory glaucoma, and experienced unbearable pain before AGV dislocation, thus we speculate that AGV tube exposure due to conjunctival dissolution could be the primary risk factor for the development of *M. houstonense* infection.

Literature states that *M. houstonense* are commonly found in water, and freshwater fish are potential reservoirs of RGM [16]. Intriguingly, the patient’s occupation was aquaculture, thus it’s concluded that the aquaculture water where he worked may be the source of RGM infection.

The diagnosis of RGM infection post-surgery is often delayed due to chronic and subtle course of disease without specific symptoms. Apart from patients with AIDS, disseminated RGM infection is usually associated with corticosteroid therapy, immunomodulating drugs and transplant patients [17]. In our case, the endophthalmitis lasted for almost 2 months without dissemination in the absence of appropriate treatment, which is probably because the patient has no any underlying diseases and endophthalmitis caused by RGM usually present as low-grade ocular inflammation [18].

Endophthalmitis is a rare complication following AGV implant surgery and results in poor vision, conjunctival erosions over the AGV tube has been reported in most cases and seem to represent a major risk factor for associated endophthalmitis [19]. The conventional treatments of endophthalmitis involve systemic and intravitreal injection of antibiotics, in combination with early pars plana vitrectomy surgery [20]. But currently, there has been no consensus on standard treatment for RGM infection. Lifelong follow-up of the eyes is very necessary to look for complications such as tube erosion after GDI surgeries, and prompt surgical intervention of conjunctival erosion is highly recommended. In patients with clinical signs and symptoms suggestive of endophthalmitis, microbiological investigations of vitreous with antibiotic susceptibility can offer rapid detection, which is of utmost importance to prevent devastating sequel of endophthalmitis.

## Data Availability

Not applicable.

## Abbreviations

RGM: Rapidly growing mycobacteria
GDI: Glaucoma drainage implant
AGV: Ahmed glaucoma valve
BCVA: Best-corrected visual acuity
MIC: Minimal inhibitory concentration

## References

[1] Brown-Elliott BA, Philley JV. Rapidly Growing Mycobacteria. Microbiol Spectr. 2017;5(1). 10.1128/microbiolspec.TNMI7-0027-2016. 10.1128/microbiolspec.tnmi7-0027-2016 PMC11687460 28084211

[2] Shah M, Relhan N, Kuriyan AE, Davis JL, Albini TA, Pathengay A (2016). Endophthalmitis caused by Nontuberculous Mycobacterium: clinical features, antimicrobial susceptibilities, and treatment outcomes. Am J Ophthalmol.

[3] Marin-Casanova P, Calandria Amiguetti JL, Garcia-Martos P, Lozano Dominguez C, Sanabria BH, Puerto Alonso JL (2003). Endophthalmitis caused by Mycobacterium abscessus. Eur J Ophthalmol.

[4] Hung JH, Huang YH, Chang TC, Tseng SH, Shih MH, Wu JJ (2016). A cluster of endophthalmitis caused by Mycobacterium abscessus after cataract surgery. J Microbiol Immunol Infect.

[5] Uy HS, Nguyen QD, Durand ML, Paton B, Foster CS (1999). Infectious crystalline keratopathy and endophthalmitis secondary to Mycobacterium abscessus in a monocular patient with Stevens-Johnson syndrome. Am J Ophthalmol.

[6] Chang V, Karp CL, Yoo SH, Ide T, Budenz DL, Kovach JL (2010). Mycobacterium abscessus endophthalmitis after Descemet's stripping with automated endothelial keratoplasty. Cornea.

[7] Benz MS, Murray TG, Dubovy SR, Katz RS, Eifrig CW (2003). Endophthalmitis caused by Mycobacterium chelonae abscessus after intravitreal injection of triamcinolone. Arch Ophthalmol.

[8] Rolfe NE, Garcia C, Widen RH, Taylor SP (2013). Rapid diagnosis of Mycobacterium abscessus endophthalmitis. J Med Microbiol.

[9] Rao A, Wallang B, Padhy TR, Mittal R, Sharma S (2013). Dual infection by streptococcus and atypical mycobacteria following Ahmed glaucoma valve surgery. Semin Ophthalmol.

[10] Suganeswari G, Shah D, Anand AR (2020). Intravitreal piperacillin-tazobactam in endophthalmitis caused by Mycobacterium abscessus in silico ne-filled eye: a case report. Indian J Ophthalmol.

[11] Valenzuela F, Oportus MJ, Perez CI, Mellado F, Cartes C, Villarroel F (2018). Ahmed glaucoma drainage implant surgery in the management of refractory uveitic glaucoma: long-term follow up. Arch Soc Esp Oftalmol.

[12] Pinitpuwadol W, Sarunket S, Boonsopon S, Tesavibul N, Choopong P (2018). Late-onset postoperative Mycobacterium haemophilum endophthalmitis masquerading as inflammatory uveitis: a case report. BMC Infect Dis.

[13] Lu X, Ng DS, Zheng K, Peng K, Jin C, Xia H (2016). Risk factors for endophthalmitis requiring evisceration or enucleation. Sci Rep.

[14] <M100ED29-2019_Decrypted(1).pdf>.

[15] Institute; CLS. Clinical and Laboratory Standards Institute (2011) Susceptibility Testing of Mycobacteria, Nocardiae, and Other Aerobic Actinomycetes; Approved Standard— Second Edition M24-A2. 2011. 31339680

[16] Lorencova A, Klanicova B, Makovcova J, Slana I, Vojkovska H, Babak V (2013). Nontuberculous mycobacteria in freshwater fish and fish products intended for human consumption. Foodborne Pathog Dis.

[17] Lopez-Luis BA, Sifuentes-Osornio J, Perez-Gutierrez MT, Chavez-Mazari B, Bobadilla-Del-Valle M, Ponce-de-Leon A (2020). Nontuberculous mycobacterial infection in a tertiary care center in Mexico, 2001-2017. Braz J Infect Dis.

[18] Garg P (2012). Fungal, mycobacterial, and Nocardia infections and the eye: an update. Eye (Lond).

[19] Al-Torbak AA, Al-Shahwan S, Al-Jadaan I, Al-Hommadi A, Edward DP (2005). Endophthalmitis associated with the Ahmed glaucoma valve implant. Br J Ophthalmol.

[20] Xu K, Chin EK, Bennett SR, Williams DF, Ryan EH, Dev S (2018). Endophthalmitis after Intravitreal injection of vascular endothelial growth factor inhibitors: management and visual outcomes. Ophthalmology.

